# Relations between indoor rehabilitation and basic health services in a developing country

**DOI:** 10.3389/fresc.2023.1001084

**Published:** 2023-01-25

**Authors:** Taslim Uddin, Badrunnessa Ahmed, Farzana Khan Shoma

**Affiliations:** Department of Physical Medicine and Rehabilitation, Bangabandhu Sheikh Mujib Medical University (BSMMU), Dhaka, Bangladesh

**Keywords:** inpatient rehabilitation, developing country, demographic profile, medical conditions, functional outcome, duration of hospital stay, mode of disposal

## Abstract

**Background:**

and Introduction: Physical rehabilitation is vital for patients to regain maximum function. Approximately 80% of people with a disability live in developing countries, where they face multiple challenges in rehabilitation. The goal of the study was to conduct an analysis of indoor rehabilitation programs based on the demographics and medical conditions of the admitted patients and to relate to the available basic health and rehabilitation facilities.

**Methods:**

This was a mixed method study conducted in an inpatient rehabilitation ward of a tertiary level academic university hospital in a developing country. All admitted patients who stayed for a period of minimum two weeks were included in the study. Demographic and clinical data were obtained by means of a retrospective medical record review utilizing a standardized data extraction form. The study was further strengthened by an online literature search for the available documents for analysis, relation, and discussion.

**Results:**

Among the 1,309 admitted patients was male- female ratio was 10:7, with the majority (31.4%) cases falling between the ages of 46 and 60yrs. Rehabilitation outpatient department was the principal mode of admission (78%), and musculoskeletal and neurological conditions represented the maximum number (79.8%). Majority of patients (60.8%) were discharged home on completion of the rehabilitation program with a large number of patients who were absconded. Poor health budget allocation and lack of prioritization of the rehabilitation sector face multiple challenges, including the rehabilitation team functioning resources, space crisis for expansion which was further impacted by the COVID-19 pandemic.

**Conclusions:**

The country's current health-related rehabilitation process and socio-demographic variables have a negative relationship. There was a large number of missing data in the medical records and many patients were lost prematurely from the indoor rehabilitation program. Musculoskeletal disorders were common, and the majority of patients were discharged home once the program was completed.

## Introduction

The World Health Organization (WHO) defines rehabilitation as a combination of interventions meant to improve functioning and decrease impairment in people with health complications (1). In some low and middle-income countries (LMIC), more than 50% of people do not receive the rehabilitation services they require (2). The need for rehabilitation worldwide is predicted to increase because of improvements in acute care services and because people are living longer. In recent years, there has been an increased demand for inpatient rehabilitation services, and rehabilitation services are also amongst the health services most severely disrupted by the COVID-19 pandemic, where expansion of services was advocated (3, 4).

Health-related rehabilitation is beneficial for all age groups to become independent of daily living activities. Physical rehabilitation is vital for patients to regain maximum function, and many of the attending patients, including stroke, spinal cord injury, traumatic brain injury, sports trauma, and postsurgical conditions, require a period of comprehensive rehabilitation in an indoor facility (5).

Health care professionals, health care facilities, medicine devices, and other technologies, including information systems and financing, are the five fundamental components for high-quality health services (6).

Following admission to a postacute rehabilitation care facility, where rehabilitation services are provided by a team of skilled professionals, it can help achieve the best possible outcome, preventing complications, facilitating recovery, optimizing medical therapy, and lowering healthcare costs (7). When patients with disabilities use more extensive rehabilitation services, including work hardening programs, in the post-acute care setting, they are more likely to be discharged to the community and make greater functional progress (8).

According to a recent survey in Bangladesh, 3.29% of men and 2.34% of women in the country have some form of disability, and most of them are physically challenged (9). Approximately 80% of the global population with disabilities lives in developing countries. Bangladesh is a lower-middle-income country with a dense population that faces numerous challenges in basic health and rehabilitation care (10).

Earlier studies of an indoor rehabilitation service described the clinical profiles of disability conditions being managed from a developing country's perspective (11). Few studies have been conducted to evaluate the effects of long-term and comprehensive inpatient rehabilitation on returning to a meaningful occupation, work hardening, gaining employment, or achieving a degree of independence in daily living activities. And there was no study available stating the demographic and medical conditions on a large scale in this kind of setting. Acute and critical care services have been addressed by the government of Bangladesh and BSMMU. However, postacute services, especially rehabilitation, were not categorized as a priority specialty. PMR was described as a fast-growing and popular specialty in Bangladesh, with a dedicated indoor facility started at BSMMU and other institutes (12). Since then, there has been a need to know about the demographic profile, nature of the conditions managed, and the functional outcome of the admitted patients in this tertiary academic hospital rehabilitation facility. There were recommendations for more research on the role of the post-acute care indoor setting and its attributes in determining health outcomes (13). The goal of the present study was to conduct an analysis of indoor rehabilitation programs in a developing country based on the demographics and medical conditions of admitted patients. It was also aimed at relating the rehabilitation process to the basic health and rehabilitation perspectives of the entity. We expect that this study will strengthen the medical information system and will assist policymakers in enacting policies to track and enhance functional results.

### Methods

Objectives: To present an analysis of the indoor rehabilitation programme and to relate the findings to the basic sociodemographic and rehabilitation status of Bangladesh.

Study design: a mixed-method study comprising a cross-sectional study and a literature review. The online literature search was conducted using PubMed, Google scholar, and Banglajol with the key words “Inpatient Rehabilitation”, “Bangladesh”, “Rehabilitation”, and “Developing Country” for the available documents for analysis and discussion.

Setting: The cross-sectional study was done in the PMR inpatient rehabilitation wards of BSMMU—a tertiary-level academic university hospital in Dhaka city during the period of June 2015 to December 2019.

Demographic and clinical data were obtained by means of a retrospective medical record review utilizing a standardized data extraction form with institutional ethical board permission. All admitted patients, both male and female, who stayed for a period of a minimum of two weeks were included in the study. Subjects who expired, could not complete the 2-week rehabilitation program, or who had an incomplete data set were excluded from the study.

### Statistical methods

Data was compiled and coded in SPSS Version 26 and analysis was done through quantitative approaches. Descriptive statistics were calculated. Categorical variables were tabulated asfrequencies and percentages and continuous variables as mean, medians, and standard deviations (SD). Prior to the main analysis, the dataset was assessed for non-normality. The results were expressed as frequency, mean, and percentage in tables, graphs, etc.

## Results

A total of 1,309 patients were admitted to the rehabilitation ward between June 2015 and December 2019. There were 770 males and 539 females with a male-to-female ratio of 10:7.

**Figure 1:**
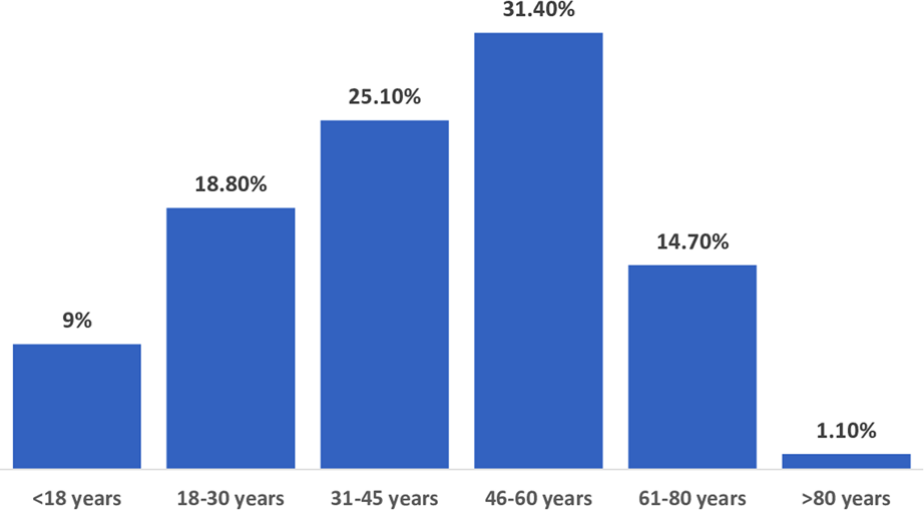
Age distribution* in years (*n* = 130).

Figure 1 depicts the age distribution of hospitalized patients over a five-year period, with the majority of 411 (31.4%) cases falling between the ages of 46 and 60 yrs.

The rehabilitation outpatient department (OPD) was the most common mode of admission (78%), and the majority of patients (60.8%) were discharged home following a period of rehabilitation. Table 1 depicts the modes of admission and discharge or disposal of the rehabilitation facility. Information on the duration of hospital stay was available in 565 (43.1%) subjects, of which a maximum number (34.4%) of patients stayed for 15–21 days.

**Table 1:** Mode of Admission and Discharge (*n* = 1309).

Mode of admission			Mode of discharge and disposal		
Parameter	Number	Percentage	Parameter	Number	Percentage
Through OPD	1023	78.15%	Discharge home	796	60.80%
Indoor Transfer	32	2.44%	Discharge on request	78	5.95%
Through emergency	18	1.37%	Discharge with risk bond	22	1.68%
Data Missing**	236	18.02	Absconded*	14	1.06%
			Data missing**	270	20.62%
			Transfer out	117	8.93%
			Expired	12	0.91%

**Figure 2:**
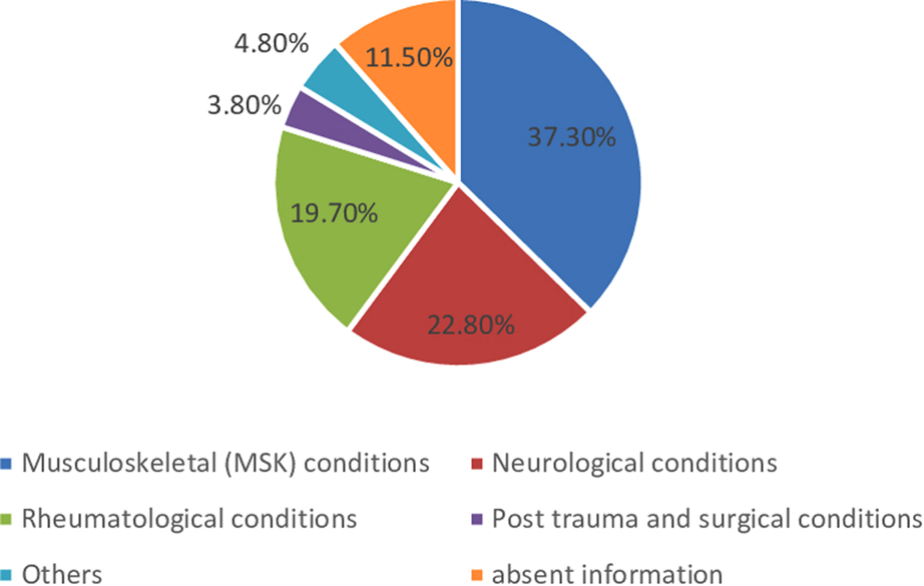
Clinical characteristics and conditions.

Figure 2 shows the clinical characteristics and diagnoses of admitted patients. Musculoskeletal conditions, neurological conditions, and rheumatological conditions constituted the maximum number (79.8%) of admissions, and there was a lower number of admissions with posttraumatic and traumatic conditions.

There is a wide gap in information about the health and rehabilitation sector in general. Poor health sector budget al.location and lack of prioritization of the rehabilitation sector face multiple challenges, including the rehabilitation team's functioning resources, infrastructure, and settings, which were further impacted by the COVID-19 pandemic. Basic health and rehabilitation indicators are placed in Box 1.

BOX 1 **Basic sociodemographic, health, and Rehabilitation indicators of Bangladesh (10, 14–16).**

– *Bangladesh – LMIC in South Asia
– population- 168,500,000, population density −1265/km^2^
– median age 27.6 years and life expectancy 72.59 years
– Per capita health care spending −42 US Dollar with 70% out of pocket expenditure
– Hospital Beds −08 hospital beds per 10,000 population
– Doctors and Nurses – 0.5 doctors and 0.2 nurses/1,000 people
– 6.8 rehabilitation unit/million people
– Rehabilitation Physicians- Total 250
– *Premedical staff including rehabilitation therapists −9.4 PT, 1.3 OT, 0.9 SLT, 0.2 P&O/million people

*LMIC-Lower-Middle -Income Country, PT-Physiotherapist, OT-Occupational Therapist, SLT-Speech language therapist and P&O-Prosthetics and orthotics

### Discussions

The WHO Rehabilitation 2030 initiative expects rehabilitation should be available at all stages of life and builds a comprehensive rehabilitation service delivery model to achieve equitable access to quality services (17). However, the speed and volume of development were slower in this country than the rehabilitation in other comparable regions of the world (18, 19).

### Mode of admission in the inpatient rehabilitation facility

Out of 1,309 patients, 1,023 (78.1%) patients were admitted through PMR OPD (Table 1), primarily screened for eligibility for admission by an associate professor or consultant assisted by a senior medical officer or resident. Most of these patients had multiple complications at admission. Many of the subjects were received with a discharge certificate from acute care facilities without referral to rehabilitation. This was in contrast to other facilities where most of the admissions for inpatient rehabilitation are from indoor transfers (20) and many of the chronic patients, including stroke survivors, were not assessed by any type of rehabilitation even in the developed world (21). This could be multifactorial, including misconceptions about physical therapy (PT) and physical rehabilitation medicine (PMR) being interchangeable terms, and many of the referring consultants are unaware of the distinctions or lack knowledge of the benefits of early rehabilitation (22).

### Mode of discharge and disposal, including absconded patients

The main goal of a course of inpatient rehabilitation is to discharge the patient back home or to the community. In this study, most of the patients (68.8%) were discharged home (Table 1) and 14 patients (1.06%) were absconded while doing inpatient rehabilitation. Discharge on request and discharge on the risk bond accounted for approximately 7.63% of the subjects, indicating that a significant number of patients left the inpatient rehabilitation program prematurely. The number of absconded patients seems to be higher in this study. The patient absconding from the hospital while on a course of treatment was described as one of the important health and security problems (23). However, the earlier studies stated absconding issues were in general hospitals and in psychiatric facilities (23, 24). We assume no health insurance and difficulty in bearing treatment costs, prolonged hospital stays without family earnings, and feeling the load of the number of routine therapy modalities and sessions while staying at this inpatient facility.

### Clinical characteristics and diagnosis of the admitted patients

Of the 727 patients examined for the broad heading diagnosis and clinical conditions, more than one-third (37.3%) of the subjects were admitted with a diagnosis of musculoskeletal (MSK) disorder (Figure 2), although many of the patients had multiple comorbidities and diagnoses. This could be due to the major MSK practice patterns of our physiatrists and less attention to other disabling conditions, including SCI and TBI (25). In some other studies, mobility was the commonest form of disability (23). However, many advanced centers in the world may not provide all categories of rehabilitation services under one roof. Some of the rehabilitation centers concentrate mostly on the regional priority conditions, for example, dedicated spinal cord injury rehabilitation, stroke rehabilitation, or orthopedic and trauma rehabilitation (26). One earlier study described diagnosis at admission as a determinant factor for better rehabilitation outcomes (27). Another important issue was that 11.5% of the patient's clinical information was missing in our study. Inadequate medical record keeping and missing clinical information are a real problem in developing countries. This was also a problem in the advanced centers, including the National Health Services in the United Kingdom (28).

### Duration of hospital stay

The length of stay and rehabilitation outcomes at a rehabilitation facility are largely governed by multiple factors (29, 30). In our study, the maximum number of patients stayed at the indoor facility for about 18 days, and this facility admitted multiple and varied conditions as stated above. A study described by the Center for Medicare Advocacy showed that the average length of stay in postacute care for all clinical categories of rehabilitation was 12.4 days (31). They admitted mostly the three clinical categories of patients, including lower extremity joint replacement (hip/knee), stroke, and hip fracture, which were infrequent in our study.

### Challenges and barriers identified for inpatient rehabilitation

The barriers and challenges for inpatient rehabilitation of this LMIC rehabilitation facility were summarized in Box 2.

BOX 2 **Challenges and barriers identified for inpatient rehabilitation.**

– timely referrals from acute care facilities
– rehabilitation team member constraints and team management difficulties
– a massive workload
– a lack of adequate time allocation for individualized or group therapy
– no insurance and universal health coverage.

## Conclusions

The current national health-related rehabilitation process and socio-demographic variables have a negative relationship. OPD provided the main source of inpatient rehabilitation. Despite a large number of missing data, MSK conditions constituted the maximum number, and the majority of the patients completed two-week programs and were discharged home. The study provided an overview of inpatient rehabilitation in an LMIC setting, which might be useful for setting rehabilitation goals and administering rehabilitation therapy modalities. and facilitating discharge planning. Indoor rehabilitation should align with the national model of health care. Further research at a multicenter based in a cross-cultural rehabilitation setting is recommended.

## Limitations of the study

The principal limitation of the study was a failure to retrieve a large number of participants' information on different characteristics, including mode of admission and discharge, and clinical characteristics.

## Data Availability

The original contributions presented in the study are included in the article/Supplementary Material, further inquiries can be directed to the corresponding author.
